# Neural correlates of motor learning: Network communication versus local oscillations

**DOI:** 10.1162/netn_a_00374

**Published:** 2024-10-01

**Authors:** Anaïs Mottaz, Branislav Savic, Leslie Allaman, Adrian G. Guggisberg

**Affiliations:** Division of Neurorehabilitation, Department of Clinical Neurosciences, University Hospital of Geneva, University of Geneva, Switzerland; SIB Text Mining Group, Swiss Institute of Bioinformatics, Carouge, Switzerland; BiTeM Group, Information Sciences, HES-SO/HEG, Carouge, Switzerland; Division of Neurorehabilitation, Department of Neurology, Inselspital, Bern University Hospital, University of Bern, Switzerland

**Keywords:** Motor sequence learning, Electroencephalography, Functional connectivity, Neural plasticity

## Abstract

Learning new motor skills through training, also termed motor learning, is central for everyday life. Current training strategies recommend intensive task-repetitions aimed at inducing local activation of motor areas, associated with changes in oscillation amplitudes (“event-related power”) during training. More recently, another neural mechanism was suggested to influence motor learning: modulation of functional connectivity (FC), that is, how much spatially separated brain regions communicate with each other before and during training. The goal of the present study was to compare the impact of these two neural processing types on motor learning. We measured EEG before, during, and after a finger-tapping task (FTT) in 20 healthy subjects. The results showed that training gain, long-term expertise (i.e., average motor performance), and consolidation were all predicted by whole-brain alpha- and beta-band FC at motor areas, striatum, and mediotemporal lobe (MTL). Local power changes during training did not predict any dependent variable. Thus, network dynamics seem more crucial than local activity for motor sequence learning, and training techniques should attempt to facilitate network interactions rather than local cortical activation.

## Author Summary

Both, local and network processing mechanisms support motor sequence learning. The aim of the present study was to compare the impact of these two processing types on motor learning. We measured EEG before, during, and after a finger-tapping task (FTT) in 20 healthy subjects. The results showed that only network dynamics, measured with functional connectivity, could predict learning, long-term expertise, and consolidation. Conversely, local activity, measured with event-related power decrease, did not predict any dependent measure. Specifically, network interactions of the primary motor area, the striatum, and the medial temporal lobe correlated with learning performance. Therefore, network dynamics seem more crucial than local activity for motor sequence learning and training techniques should facilitate network interactions rather than local cortical activation.

## INTRODUCTION

Motor learning is central for daily life, from when we are born until elderly age. Seminal studies applied electroencephalography (EEG) during training and showed that spontaneous oscillation amplitudes in the alpha (8–12 Hz) and beta (13–30 Hz) bands decreased as soon as participants expressed a motor action (Pfurtscheller et al., 1996). As convergent results suggest that alpha activity is directly proportional to inhibitory mechanisms (Haegens et al., 2011; Jensen & Mazaheri, 2010; Klimesch, 2012), a decrease in alpha power indicates a reduction of inhibitory processing favoring, in turn, activation. Moreover, the reduction of beta power seems to reflect a high computational load specifically on the motor cortex needed when learning new movements (Baker et al., 1999). Thus, these event-related power changes in alpha and beta bands are considered metrics of local cortical activity of motor areas (Pfurtscheller & Lopes da Silva, 1999). Modulation of local cortical activity of the motor cortex correlates with motor performance (Boonstra et al., 2007; Khanna & Carmena, 2017) and learning (Espenhahn et al., 2019; Pollok et al., 2014). Therefore, current training strategies mostly focus on inducing maximal activation of motor areas through intensive, challenging but feasible repetitions of useful skills (Kleim & Jones, 2008; Langhorne et al., 2011).

More recent data processing methods allow investigating neural interactions between spatially separated brain regions. Cross-regional neural interactions are associated with a synchronization of neural oscillations, that is, with functional connectivity (FC) between them (Fries, 2005). FC during tasks seems one of the main neural mechanisms supporting cognitive performance. A growing number of studies show that FC during resting-state, that is when participants are not engaged in an explicit task, can predict motor, perceptual, and cognitive performance (Fries, 2005, 2015; Gentili et al., 2015; Guggisberg et al., 2015; Koyama et al., 2011; Sadaghiani et al., 2015; Siegel et al., 2012; Stam & van Straaten, 2012; Wu et al., 2014). During rest, our brain preferentially seems to use FC in the alpha and beta frequency bands for interregional communication (Chapeton et al., 2019; Guggisberg et al., 2008, 2015). Allaman et al. (2020) showed that behavioral performance in visual detection and motor planning tasks is best in those participants who had high global alpha-band resting-state FC between the involved brain area (visual or motor cortex, respectively) and the rest of the brain, but low local activation (as indexed by low event-related power change) during the task. This suggests that resting-state neural interactions allow for a more efficient task performance than classical local activations. Recent neuroimaging studies also suggest that task FC plays a critical role for cognitive task performance (Cole et al., 2021; Ito et al., 2020).

Resting-state FC seems to be not only associated with better current task performance, but also with better learning (Albert et al., 2009; Bonzano et al., 2015; Gregory et al., 2014). Indeed, several studies showed that resting-state FC in alpha and beta frequency bands predicts motor learning (Manuel et al., 2018; Mary et al., 2017; Van Dyck et al., 2021; Wu et al., 2014). Previous work also demonstrated a relationship between beta-band FC and motor recovery in the first months after stroke (Nicolo et al., 2015). Some studies have also linked task FC dynamics with learning (Bassett et al., 2011; Tóth et al., 2017).

In summary, local and network activity measured through event-related power changes and FC, respectively, are two neural mechanisms supporting motor learning. However, since earlier studies considered these two mechanisms separately, their relative contribution to motor learning is unknown. Here, we used EEG to quantify both event-related power changes and alpha- and beta-band FC as predictors of learning. Specifically, we measured EEG before, during, and after a motor sequence learning task that is the finger tapping task (FTT) (Walker et al., 2002). Based on previous results and current models of motor learning (Allaman et al., 2021; Doyon et al., 2009; Manuel et al., 2018), we hypothesized that alpha- and beta-band FC at rest, alpha- and beta-band FC during training, and event-related power changes during training would predict learning.

## METHODS

### Participants

We recruited 20 healthy right-handed participants with no history of neurologic or psychiatric disorders (13 women; 28.7 ± 5.6 years old). This sample size was chosen according to previous studies that found correlation coefficients of about 0.6 between functional connectivity and behavior (e.g., Allaman et al., 2020; Guggisberg et al., 2015), giving us >80% power to detect similar associations at *p* < 0.05. After receiving an explanation of the experiment, all participants gave written informed consent. They all received monetary compensation for participation. Additionally, they were asked whether they had experience with playing the piano and, if yes, for how many years they practiced. The ethical committee of the Canton of Geneva approved the experimental procedure performed according to the Declaration of Helsinki.

### Experimental Procedure

We applied a finger tapping task (FTT) as described in the literature (Alger et al., 2017). The same sequence was used for all participants to avoid task-related variability, which might confound with the neural variability that was of interest here. Participants were instructed to repeat a given five-item sequence with their left hand (little finger to index) on four horizontally arranged buttons numbered left to right on a Chronos box (Psychology Software Tools; https://pstnet.com/products/chronos/). The same sequence was trained throughout the whole experiment (1-4-2-3-1) and designed using E-Prime 2.0 (Psychological Software Tools). It was continuously presented to participants while they had to perform it. Continuous EEG was recorded using a 128-channel BioSemi ActiveTwo EEG-system (BioSemi B.V.) with a sampling rate of 1024 Hz.

**Figure 1.**
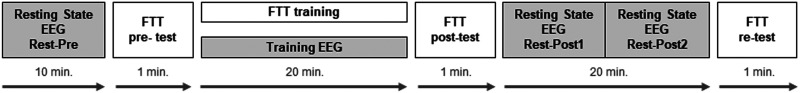
Experimental procedure. Gray boxes represent EEG data acquisition; white boxes FTT blocks.

Figure 1 depicts a schematic representation of the procedure. The experiment started with 10-min eyes-closed resting-state. The instruction for these 10 minutes were to sit comfortably, relax as much as possible, and not fall asleep. Then, participants performed four blocks of the FTT: a *pre-test* block, a *training* block, a *post-test* and a *re-test* block. Between the post-test and re-test blocks, 20 minutes of eyes-closed resting-state EEG were acquired. During test blocks, participants had to repeat the five-element sequence with their nondominant left hand (little finger to index) as fast and accurately as possible during two trials of 30 seconds (s). Participants had a break of 30 s between these two trials. The training block was designed to analyze neural activity time-locked to the beginning of the movement (see subsection Training Task FC). It contained 150 trials. Each trial was composed of two consecutive sequences, to allow training the transition between sequences. The start of the trials was cued with the apparition of the sequence on the screen, and a feedback was given with a star appearing under each element correctly pressed. In case of error, participants had to repeat the element until correct. A delay of 1.5 s between trials was set (last keypress to next sequence) to minimize the postmovement neural effect on the following sequence repetition.

### Behavioral Analyses

The main dependent behavioral variable was the number of correctly completed sequences in a given test block (i.e., mean number of correctly completed sequences in the two combined 30-s trials). *Learning* was defined as the difference between post- and pre-test blocks performances. *Long-term skill expertise* was measured as the average performance across pre-test, post-test, and re-test blocks. *Consolidation* was measured as the difference between re- and post-test blocks performances.

### EEG Source Localization

Artefacts, such as eye movements, blinks, power line, electrode and muscular artefacts were removed by visual inspection—removing noisy data segments—for resting-state data and using independent component analysis (FastICA algorithm) (Hyvärinen, 1999) for data recorded during the training task, removing the components according to their aspect in the time series and their scalp topography. Electrodes that were noisy along most of the signal were removed from the data.

Source imaging was performed in MATLAB (The MathWorks), using the toolbox NUTMEG (Dalal et al., 2011) and its Functional Connectivity Mapping toolbox (Guggisberg et al., 2011). Lead-potential was computed using a boundary element head model, with the Helsinki BEM library (Stenroos et al., 2007) and the NUTEEG plugin of NUTMEG (Guggisberg et al., 2011). The head model was based on the individual T1 MRI of each participant, and solution points were defined in the gray matter with 10-mm grid spacing. EEG data were band-pass filtered in the alpha (8–12 Hz) and beta (13–30 Hz) frequency bands, Hanning windowed, Fourier transformed, and projected to gray matter voxels using an adaptive filter (scalar minimum variance beamformer) (Sekihara et al., 2004).

Regions of interest (ROIs) were defined anatomically. Consistent with previous findings on FTT and current models of motor sequence learning (Albouy et al., 2008, 2012; Doyon et al., 2003, 2009; Lehéricy et al., 2005), we a priori defined the following three anatomical ROIs: right primary motor and dorsolateral premotor cortex, right striatum (putamen and caudate nucleus), and right MTL (hippocampus and parahippocampus). We used the Mars Atlas for defining the motor area (Auzias et al., 2016) and the automated anatomical labelling (AAL) atlas for striatum and MTL (Rolls et al., 2020).

### Resting-State FC

The artifact-free resting-state data were segmented into 300 nonoverlapping 1-s epochs. FC was estimated as the absolute imaginary component of coherency (IC), as described previously (Guggisberg et al., 2011, 2015). Its value depends on the stability of the phase difference across time windows. IC is a spectral measure of FC ignoring zero-phase lag coherence (Nolte et al., 2004), making it more robust to spurious or biased interactions due to volume conduction or spatial leakage of the inverse solution (Sekihara et al., 2011). We calculated the global FC of each voxel as weighted node degree (WND), that is, as the sum of its IC with all other cortical voxels (Guggisberg et al., 2015; Mottaz et al., 2018; Newman, 2004). WND quantifies how much a specific area is important for the whole brain network (Stam & van Straaten, 2012). It abstracts from the interactions with specific other areas, but simply informs out the overall communication of a brain area. This allows for interindividual differences in the precise architecture of the functional network and also captures to some degree interactions across several brain regions. FC values can be influenced by the signal-to-noise ratio of the EEG. To minimize this potential confound, we normalized WND values by calculating Z-scores. This was achieved by subtracting the mean WND value of all voxels of the subject from the WND values at each voxel and by dividing by the standard deviation (SD) over all voxels (Guggisberg et al., 2011; Mottaz et al., 2015). ROI FC values were obtained by averaging the normalized WND of the corresponding voxels.

We focused our analysis of FC on the alpha and beta frequency bands, as several studies showed that resting-state FC in these frequencies predicts motor learning (Manuel et al., 2018; Mary et al., 2017; Van Dyck et al., 2021; Wu et al., 2014). Moreover, alpha is the main frequency for resting interactions (Chapeton et al., 2019).

### Training Task FC

To investigate the time course of neural interactions during training, we used an event-related analysis of FC (Andrew & Pfurtscheller, 1996). Event-related imaginary coherence (ERImCoh) has been shown to be a good metric of stimulus-induced FC (Yoshinaga et al., 2020). It was computed similarly as resting-state FC, except that coherence values were obtained by summing across trials of the FTT training instead of time windows, thus enabling a better temporal resolution. On average, there were 114 clean trials per subject corresponding to the repetitions of the pairs of sequences. To obtain a similar number of trials across participants, we selected and analyzed a subset of 100 trials based on the alpha to other frequency band amplitude ratio, aiming to choose trials that exhibited minimal noise from muscles or eye blinks. For each trial, we segmented the preprocessed data into time windows of 125 ms. To account for differences in reaction time between participants, we fixed three time windows on specific events: the appearance of the cue, the first keypress, and the last keypress. Between these events, we defined the same number of time windows for each participant, spaced at regular intervals. The overlap of these time windows varied according to individual reaction times to ensure that the total number of time windows was the same for all participants and aligned with the three key events.

In addition, we computed FC during baseline by averaging ERImCoh values between 625 ms and 375 ms before sequence cue apparition. ERImCoh at each active time window was then computed as the difference between FC during training and baseline. Final value of FC training was obtained by averaging ERImCoh values of time windows between the first and last keypress.

### Event-Related Power Changes

Task-induced power modulations during training were computed as the average root-mean-square of the 125-ms time windows at the source level. Power was averaged across the 100 artifact-free epochs for each participant and the baseline was subtracted.

The event-related power band decomposition was first computed to check for the reliability of the power data.

Final value of power in the alpha and beta band during training was calculated by averaging power values of time windows between the first and last keypress, as for FC. To approach normal distribution, we log-transformed the power values.

### Statistical Analyses

All statistical analyses were performed using the Statistics Toolbox of MATLAB 2018b. The data used in the present study will be made available upon request due to the need for approval from the requesting researcher’s local ethics committee.

To evaluate motor learning and consolidation, a repeated measures analysis of variance (RM-ANOVA) was performed on the mean number of correctly completed sequences per minute with test block (pre-test vs. post-test vs. re-test) as within-subject factor. A level of significance of *p* < .05 (two-tailed) was used. Effect sizes are indicated in *η*^2^.

In order to adjust for multiple comparisons and to find the independent predictors of learning, long-term expertise, and consolidation from both local and network measures in a multivariate approach, we used stepwise linear regression. Specifically, we used forward selection (*p* value threshold of 0.06) followed by backward elimination (*p* value threshold of 0.1) based on the *p* values for *F* test of the change in the sum of squared error to identify predictors. The statistical level of significance was *p* < .05 (two-tailed). The effect sizes were quantified in standardized beta coefficients. For learning as a dependent variable, the following predictors were considered: alpha- and beta-FC Rest-Pre, alpha- and beta-FC Training, event-related power changes (alpha-Power Training and beta-Power Training), alpha- and beta-FC Rest Change (Post1 minus Rest) at the three ROIs (motor areas, striatum, and MTL). For long-term skill expertise, the following predictors were considered: alpha- and beta-FC Rest-Pre, alpha- and beta-FC Training, event-related power changes (alpha-Power Training and beta-Power Training) at the three ROIs (motor areas, striatum, and MTL). For consolidation, the following variables were considered: alpha- and beta-FC Training, event-related power changes (alpha-Power Training and beta-Power Training), alpha- and beta-FC Rest Change (Post1 minus Pre), alpha- and beta-FC Rest-Post Change (Post2 minus Post1), at each of the three ROIs (motor areas, striatum, and MTL).

All variables were checked for the presence of outliers using a median absolute difference rule (Hall & Welsh, 1985). If outliers were present or data did not meet the assumptions of normality, values were rank transformed. Since experience shapes synaptic architecture that in turn influences both resting-states and how tasks are processed (Allaman et al., 2020; Weisz et al., 2014), we assessed participants’ motor experience by asking them whether they played the piano and, if yes, for how many years. We used years of piano playing to adjust the models.

## RESULTS

### Behavioral Results

**Figure 2.**
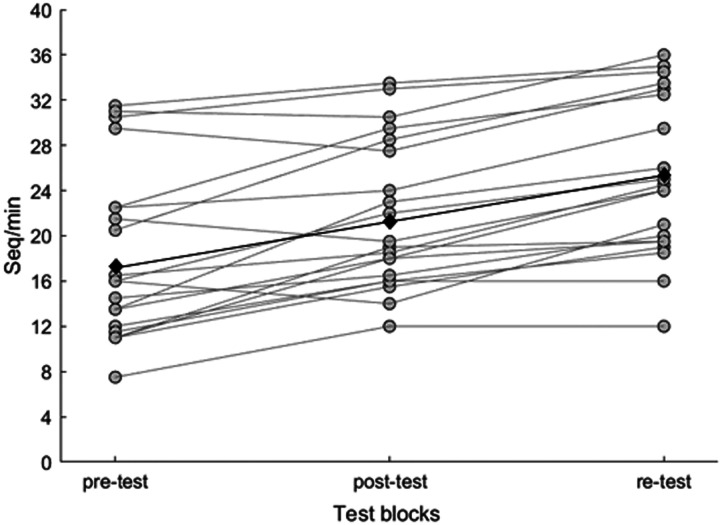
Behavioral learning in the FTT. Correctly completed sequences per minute for each test block (pre-test vs. post-test vs. re-test) and participant. Gray dots and black diamonds represent each participant and grand average, respectively.

Figure 2 shows the number of sequences per minute for each test block (pre-test, post-test, and re-test) and participant. The RM-ANOVA showed a significant effect of the within-subject factor test block (*F*_2, 38_ = 56.104, *p* < .001, *η*^2^ = 0.747). Further *t* tests showed that participants performed more sequences per minute from pre-test (*M* = 18.18, *SD* = 7.60) to post-test (*M* = 21.73, *SD* = 6.59, t_19_ = −4.56, *p* < .001) and from post-test (*M* = 21.73, *SD* = 6.59) to re-test (*M* = 25.15, *SD* = 7.10, t_19_ = −7.15, *p* < .001), indicating that sequence learning and consolidation took place.

### Event-Related Power

**Figure 3.**
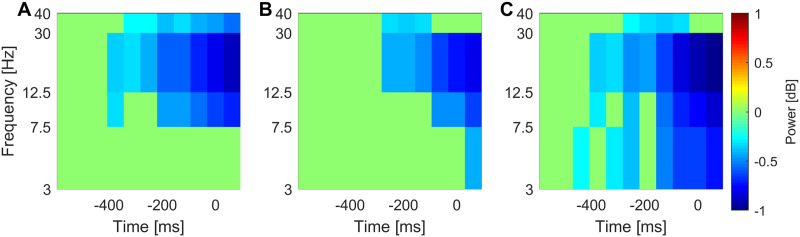
Event-related power change during training. (A) Motor ROI, (B) striatal ROI, and (C) MTL ROI. The time point zero indicates the button press.

Figure 3 shows the average event-related power change during learning. We can observe the presence of the alpha- and beta-band desynchronization during training.

### Predictors of Learning

**Figure 4.**
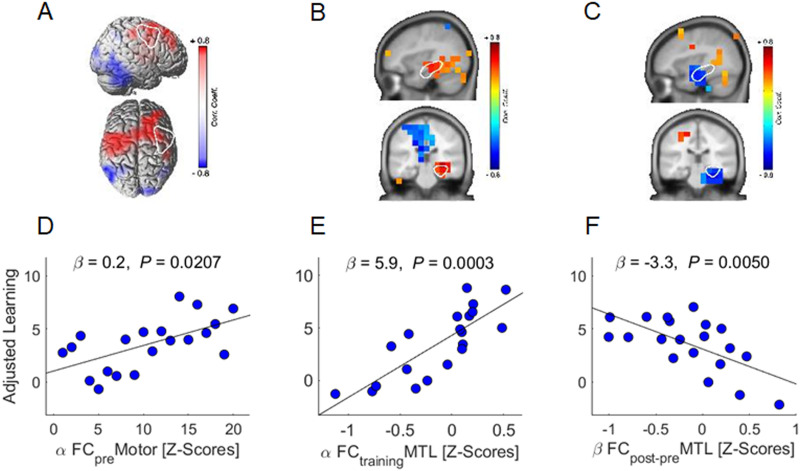
Neural predictors of learning. (A–C) Voxel-wise correlation with white overlay of ROIs (i.e., motor and MTL) cutout. (D–F) Regression plots illustrating the significant correlations of the multivariate analysis.

Table 1 shows the output of the multivariate stepwise linear regression model run on learning. The analyses showed that better learning was predicted by higher alpha-band FC in motor areas during the resting-state pre-training (Figure 4D) (*β* = 0.2, *p* = .021) and higher alpha-band FC of the MTL during training (Figure 4E) (*β* = 5.9, *p* < .001). Additionally, the decrease in resting-state beta-band FC in the MTL from before to after training predicted learning (Figure 4F) (*β* = −3.3, *p* = .005). Notably, neither alpha nor beta event-related power changes during training predicted learning (*p* > 0.14).

**Table 1.** Predictors of learning

	Regression Statistics				
	*R-Squared*	*Adjusted R-Squared*	*Root Mean Squared Error*	*Observations*	*Error degrees of freedom*
Learning ∼ 1 + alphaFCRestPreMotor + alphaFCTrainingMTL + betaFCRestChangeMTL	0.69	0.63	2.11	20	16
	ANOVA				
	*Sum of squares*	*Degrees of freedom*	*Mean squares*	*F*	*p Value*
**alphaFCRestPreMotor**	29.38	1	29.38	6.58	0.021
**alphaFCTrainingMTL**	96.49	1	96.49	21.62	<0.001
**betaFCRestChangeMTL**	47.23	1	47.23	10.58	0.005
Error	71.4	16	4.46	1	0.5
	Coefficients				
	*Estimate*	*Standard Error*	*t Stat*	*p Value*	
(Intercept)	1.32	1.04	1.26	0.22	
**alphaFCRestPreMotor**	0.24	0.09	2.57	0.021	
alphaFCRestPreStriatum	0.14	0.09	1.53	0.15	
alphaFCRestPreMTL	0.63	1.18	0.53	0.6	
alphaFCRestChangeMotor	1.12	1.41	0.79	0.44	
alphaFCRestChangeStriatum	−2.77	1.44	−1.92	0.07	
alphaFCRestChangeMTL	−0.01	0.09	−0.16	0.88	
alphaFCTrainingMotor	0.1	0.99	0.1	0.92	
alphaFCTrainingStriatum	−1.86	1.4	−1.33	0.2	
**alphaFCTrainingMTL**	5.91	1.27	4.65	<.001	
betaFCRestPreMotor	−0.02	0.09	−0.22	0.83	
betaFCRestPreStriatum	0.82	1.26	0.65	0.53	
betaFCRestPreMTL	0.18	1.02	0.17	0.87	
betaFCRestChangeMotor	−0.01	0.1	−0.08	0.94	
betaFCRestChangeStriatum	−0.05	0.1	−0.52	0.61	
**betaFCRestChangeMTL**	−3.29	1.01	−3.25	.005	
betaFCTrainingMotor	−0.78	1.22	−0.64	0.53	
betaFCTrainingStriatum	−0.03	0.09	−0.38	0.71	
betaFCTrainingMTL	0.52	2.68	0.2	0.85	
alphaPowerTrainingMotor	0.31	0.56	0.55	0.59	
alphaPowerTrainingStriatum	0.13	1.13	0.12	0.91	
alphaPowerTrainingMTL	1.34	0.91	1.47	0.16	
betaPowerTrainingMotor	0.41	0.55	0.74	0.47	
betaPowerTrainingStriatum	0.05	0.09	0.54	0.6	
betaPowerTrainingMTL	1.65	1.05	1.57	0.14	
Years Piano	−0.16	0.14	−1.16	0.26	

*Note*. Complete output of the stepwise linear regression run on learning. For ease, significant predictors are highlighted in bold.

### Predictors of Long-Term Expertise

**Figure 5.**
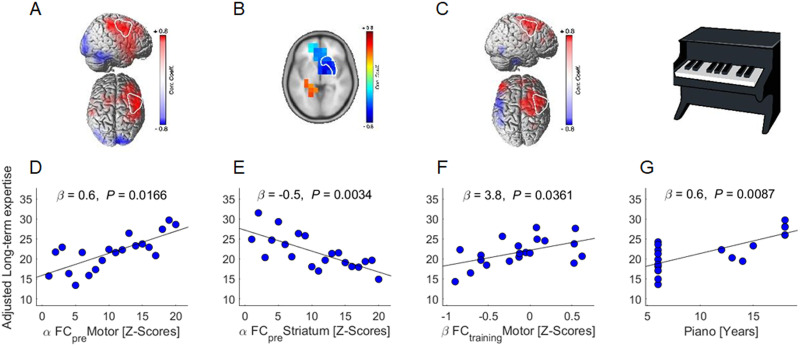
Neural predictors of long-term expertise. (A–C) Voxel-wise correlation with white overlay of ROIs (i.e., motor and striatum) cutout. (D–G) Regression plots illustrating the significant correlations of the multivariate analysis.

Table 2 shows the output of the multivariate stepwise linear regression model run on long-term skill expertise. Better long-term skill expertise was predicted by higher alpha-band FC in motor areas during the resting-state pre-training (Figure 5D) (*β* = 0.6, *p* = .017), less alpha-band FC in the striatum during resting-state pre-training (Figure 5E) (*β* = −0.5, *p* = .003), higher beta-band FC in motor areas during FTT training (Figure 5F) (*β* = 3.8, *p* = .036), and more years of piano playing (Figure 5G) (*β* = 0.6, *p* = .009). Alpha and beta event-related power changes during training were not significant predictors (*p* > 0.13).

**Table 2.** Predictors of long-term skill expertise

	Regression Statistics				
	*R-Squared*	*Adjusted R-Squared*	*Root Mean Squared Error*	*Observations*	*Error degrees of freedom*
Long-term skill expertise ∼ 1 + alphaFCRestPreMotor + alphaFCRestPreStriatum + betaFCTrainingMotor + YearsPiano	0.82	0.77	3.33	20	15
	ANOVA				
	*Sum of squares*	*Degrees of freedom*	*Mean squares*	*F*	*p Value*
**alphaFCRestPreMotor**	80.63	1	80.63	7.27	0.017
**alphaFCRestPreStriatum**	133.83	1	133.83	12.07	0.003
**betaFCTrainingMotor**	58.75	1	58.75	5.3	0.036
**YearsPiano**	100.79	1	100.79	9.09	0.009
Error	166.37	15	11.09	1	0.5
	Coefficients				
	*Estimate*	*Standard Error*	*t Stat*	*p Value*	
(Intercept)	15.29	2.15	7.11	0	
**alphaFCRestPreMotor**	0.55	0.21	2.7	0.017	
**alphaFCRestPreStriatum**	−0.52	0.15	−3.47	0.003	
alphaFCRestPreMTL	−0.42	2.1	−0.2	0.84	
alphaFCTrainingMotor	−0.71	1.66	−0.43	0.68	
alphaFCTrainingStriatum	−3.57	2.73	−1.31	0.21	
alphaFCTrainingMTL	0.09	2.28	0.04	0.97	
betaFCRestPreMotor	0.09	0.15	0.56	0.58	
betaFCRestPreStriatum	0.48	1.32	0.36	0.72	
betaFCRestPreMTL	0.11	1.48	0.07	0.94	
**betaFCTrainingMotor**	3.83	1.66	2.3	0.036	
betaFCTrainingStriatum	−0.07	0.17	−0.42	0.68	
betaFCTrainingMTL	−0.23	2.98	−0.08	0.94	
alphaPowerTrainingMotor	−1.43	0.9	−1.59	0.13	
alphaPowerTrainingStriatum	−1.95	1.84	−1.06	0.31	
alphaPowerTrainingMTL	0.3	1.55	0.2	0.85	
betaPowerTrainingMotor	−1.15	0.92	−1.25	0.23	
betaPowerTrainingStriatum	0.02	0.14	0.11	0.91	
betaPowerTrainingMTL	1.41	1.64	0.86	0.4	
**YearsPiano**	0.63	0.21	3.01	0.009	

*Note*. Complete output of the stepwise linear regression run on long-term expertise. Highlighted in bold, the significant predictors.

### Predictors of Consolidation

**Figure 6.**
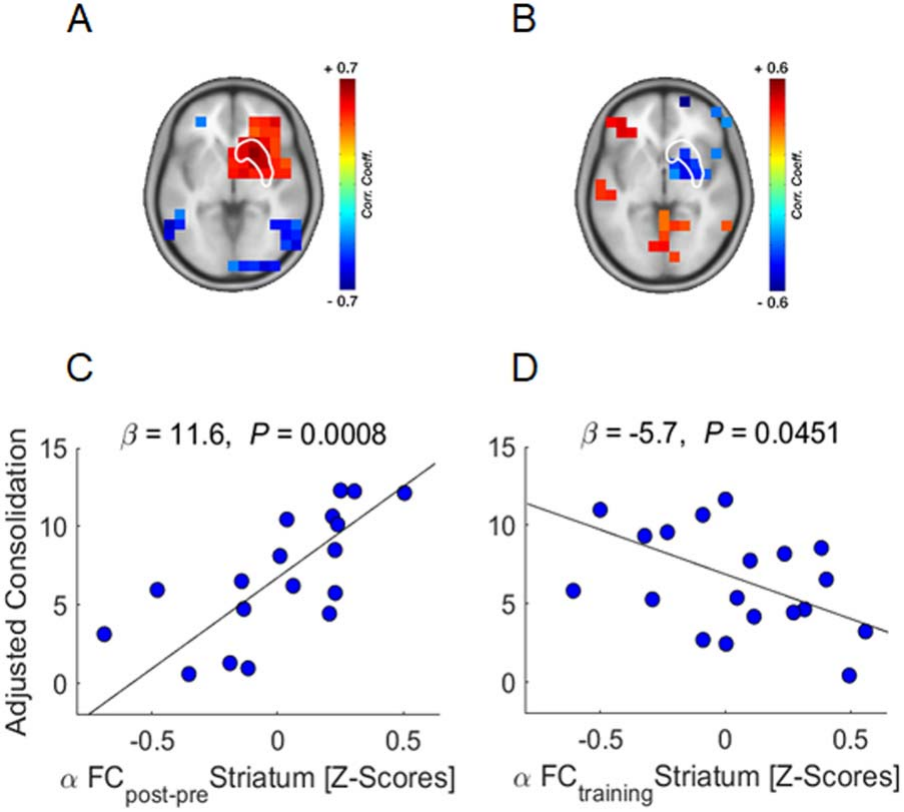
Neural predictors of consolidation. (A–B) Voxel-wise correlation with white overlay of ROI (i.e., striatum) cutout. (C–D) Regression plots illustrating the significant correlations of the multivariate analysis.

Table 3 shows the output of the multivariate stepwise linear regression model on consolidation. The increase in resting-state FC of the right striatum from before to after learning (alpha-FC Rest Change) correlated with consolidation (*β* = 11.6, *p* = .001) (Figure 6C). In addition, the decrease in striatum FC during training (*β* = −5.7, *p* = .045) predicted consolidation (Figure 6D). Neither the modulation of power during training (alpha- and beta-Power Training) (*p* > .66), nor FC change during the 20 minutes resting-state post (alpha-FC Rest-Post Change) (*p* > .58), nor years of piano playing (*p* = .76) were found to predict consolidation.

**Table 3.** Predictors of consolidation

	Regression Statistics				
	*R-Squared*	*Adjusted R-Squared*	*Root Mean Squared Error*	*Observations*	*Error degrees of freedom*
Consolidation ∼ 1 + alphaFCRestChangeStriatum + alphaFCTrainingStriatum	0.49	0.43	3.22	20	17
	ANOVA				
	*Sum of squares*	*Degrees of freedom*	*Mean squares*	*F*	*p Value*
**alphaFCRestChangeStriatum**	170.02	1	170.02	16.38	0.001
**alphaFCTrainingStriatum**	48.57	1	48.57	4.68	0.045
Error	176.49	17	10.38	1	0.5
	Coefficients				
	*Estimate*	*Standard Error*	*t Stat*	*p Value*	
(Intercept)	6.79	0.72	9.42	0	
alphaFCRestChangeMotor	1.32	1.84	0.72	0.48	
**alphaFCRestChangeStriatum**	11.58	2.86	4.05	0.0008	
alphaFCRestChangeMTL	0.08	2	0.04	0.97	
alphaFCTrainingMotor	1.82	1.55	1.17	0.26	
**alphaFCTrainingStriatum**	−5.68	2.63	−2.16	0.045	
alphaFCTrainingMTL	−0.81	1.84	−0.44	0.67	
alphaPowerTrainingMotor	0.17	0.93	0.19	0.85	
alphaPowerTrainingStriatum	0.21	1.73	0.12	0.9	
alphaPowerTrainingMTL	−0.54	1.49	−0.36	0.72	
betaPowerTrainingMotor	0.41	0.91	0.45	0.66	
betaPowerTrainingStriatum	0.74	1.74	0.43	0.68	
betaPowerTrainingMTL	−0.2	1.72	−0.11	0.91	
alphaFCRestPostChangeMotor	1.02	1.83	0.56	0.58	
alphaFCRestPostChangeStriatum	−0.55	2.04	−0.27	0.79	
alphaFCRestPostChangeMTL	−0.25	1.79	−0.14	0.89	
YearsPiano	0.05	0.15	0.31	0.76	

*Note*. Complete output of the stepwise linear regression run on consolidation.

Given the limited spatial resolution of EEG in particular for deep brain areas, we verified that results are not due to spatial leakage between sources. All correlations implying Striatum or MTL were still significant when removing shared variance of the other region of interest with a partial correlation (*p* < 0.026), confirming that both sources have distinct contribution to the source-localized EEG.

## DISCUSSION

To the best of our knowledge, this is the first study aimed at assessing the impact of local activity (i.e., event-related power changes) and network activity (i.e., FC) on motor sequence learning. Our results show that only network activity, but not local activation, predicts learning. These results are consistent with models suggesting the critical role of networks (e.g., fronto-striatal networks) rather than local activity for motor sequence learning (see Doyon et al., 2009), while they challenge the classical notion according to which local cortical activation needs to be maximized for training gains.

For example, noninvasive brain stimulation often intends to boost training gains in a specific task by increasing local activity of a cortical region. Still, brain stimulation techniques frequently do not influence behavior (Dedoncker et al., 2016; Goldsworthy et al., 2021; Horvath et al., 2015; Savic et al., 2019), and neurophysiological effects often do not translate into behavioral improvements (Masina et al., 2021). Previous work highlighted the importance of taking into account network activity when planning or interpreting brain stimulation outcomes (Bikson & Rahman, 2013; Fertonani & Miniussi, 2017; Miniussi et al., 2013). The present results corroborate this point of view and more generally show that, when aiming at increasing training gains with any kind of intervention, targeting network activity will likely lead to greatest behavioral effects.

Numerous previous studies have investigated brain activity during FTT by means of neuroimaging, creating models on the neurobiological basis of motor sequence learning (Dayan & Cohen, 2011; Doyon et al., 2009). In particular, motor areas show enhanced inter- and intrahemispheric connectivity during the beginning stages of the FTT (Sun et al., 2007). This enhanced connectivity of motor regions seems to indicate fast transformations of the spatial and motor features of the sequence (Hikosaka et al., 2002). This is in agreement with our finding that FC of motor areas predicted learning in the FTT.

Models based on neuroimaging findings proposed that FTT performance across multiple sessions are associated with (1) activation of motor areas and (2) a decrease in activation of the associative parts of the striatum that reflects a shift to the sensorimotor parts (Coynel et al., 2010; Doyon et al., 2009; Poldrack et al., 2005). Our results showing a positive correlation between long-term expertise and FC in motor areas at rest and during training are in line with these models. Furthermore, the negative correlation between long-term expertise and striatal FC at rest might be the decrease in activity of the associative parts of the striatum documented in previous models.

Despite the fact that we observed alpha- and beta-band power decrease during training (see Figure 3), local activation did not predict performance in the FTT. This is not surprising given that other studies failed to show that movement-related beta power predicts motor learning (Moisello et al., 2015; van der Cruijsen et al., 2021). Hence, the relationship between modulation of local cortical activity and motor learning is not as obvious as one might expect.

The aforementioned models also showed that MTL activity predicts motor sequence learning independently of how much participants are conscious about the sequences (Doyon et al., 2009; Schendan et al., 2003). Because of the MTL’s role for spatial and temporal processing (Reddy et al., 2021; Ruiz et al., 2020), MTL activity might reflect a better association of temporally discontinuous but structured information as in the case of a sequence (Kumaran & Maguire, 2006; Schendan et al., 2003). Statistical learning of continuous sequences depends indeed on a functional MTL (Ruiz et al., 2020) and previous work speculated on the MTL’s role in creating allocentric maps (i.e., finger movements in space) (Albouy et al., 2012). Our findings are in accordance with these models by showing that MTL FC during training predicted learning. Moreover, the present data showed that more learning was associated with a bigger MTL FC reduction from before to after training. This suggests that global brain interactions reduce while expertise increases (Percio et al., 2010) as seen in previous EEG (Gentili et al., 2015; Manuel et al., 2018; Nicolo et al., 2015) and functional imaging studies (Sami et al., 2014; Tzvi et al., 2015). In other words, training induces a specialization of motor pathways reflected in a reduction of global FC across the brain in favor of more selected interactions between brain areas that are relevant for the task.

Although it was not the main focus of the study, we measured the transformation of memory taking place after learning, namely memory consolidation, by repeating the FTT after a period in which participants were not exposed to the task (Dudai et al., 2015; Robertson et al., 2004). Previous research showed that the MTL has an established role in sleep-dependent memory consolidation (Dudai et al., 2015; Goto et al., 2021; Klinzing et al., 2019; Tonegawa et al., 2018). In the present data local activity and FC of the MTL did not correlate with memory consolidation. This may be because we measured memory consolidation 30 minutes after training and not after a night of sleep as in previous studies (Diekelmann et al., 2009; Robertson et al., 2004; Stickgold, 2005).

Previous results further showed that striatal activity during training predicts memory consolidation (Albouy et al., 2012; Peigneux et al., 2003). The striatum is thought to be involved in automatizing the motoric sequential response in the FTT (Peigneux et al., 2000). In our study, not local striatum activation, but the enhancement of network activity of the striatum from before to after training was associated with consolidation. This underlines the behavioral advantage of network processing, also for consolidation.

Striatal FC during training showed a negative correlation with consolidation. This may suggest that the changes in FC taking place during training are not as beneficial as the changes in resting-state FC. Further evidence is needed to confirm whether resting-state network changes are more impactful on consolidation than training network changes.

Experience shapes synaptic architecture that in turn influences both resting-states and how tasks are processed (Allaman et al., 2020; Weisz et al., 2014). We used years of piano playing as a measure of motor experience. The results indicated that participants with the highest average sequences per minutes (i.e., long-term expertise) across test blocks were those with more years of piano playing. In addition, our findings suggest that experience is expressed in the global level of interaction of primary motor areas and the striatum.

Thus, the present work showed that resting-state FC can predict learning, long-term expertise, and consolidation. There are several models about the role of resting-state FC for behavior (see Harmelech & Malach, 2013, for review). Some of them proposed that more FC at rest indicates that the brain has more resources (e.g., more neurons close to action potential or more computational solutions) to be used in the tasks at hand (Amit, 1989; van Vreeswijk & Sompolinsky, 1996). Others proposed that FC at rest indicates an efficient use of neural resources (Haier et al., 1992), or a neural network that is better at predicting the events that are going to take place (Euler, 2018). All these models do not necessarily exclude one another, and it can be that each model is more or less valid for specific brain regions and individuals.

Our analyses concentrated on global whole-brain connectivity as quantified with WND. Yet, pairwise FC between ROIs may be relevant as well. We actually did try to include pairwise FC between ROIs in previous predictive models. In no case did we observe that a pairwise FC was preferred over global WND in the stepwise regression. We believe that this is because WND allows for individually different network architectures and informs also about multivariate interactions across several brain regions. Thereby, WND seems to provide more useful information for predicting learning.

A debated topic in the field is the nonperiodic signal that permeates the back of EEG recordings (Gerster et al., 2022). Typically, healthy subjects display a clear FC peak in alpha frequencies at rest (Guggisberg et al., 2008, 2015), which is one of the reasons we primarily looked at the alpha band. Since converging evidence from different studies show that beta frequencies are important for learning (Nicolo et al., 2015; Wu et al., 2014), we added beta band in our analyses as well. There is ample evidence for periodic alpha and beta oscillations in the motor system (Aumann & Prut, 2015; Engel & Fries, 2010; Schnitzler et al., 2000).

The present study has limitations. From our neuroimaging results, we can only show correlations and not make inferences on causality. Initial studies have started to provide evidence with neuromodulation that increases in network coupling leads to behavioral gains (Mottaz et al., 2018), but this needs to be confirmed more formally for learning. Moreover, the spatial resolution of EEG source analyses is limited, especially for deeper brain regions such as the striatum and the MTL. The ability of EEG to localize signals coming from subcortical areas is therefore debated (Andersen et al., 2020; Attal & Schwartz, 2013; Krishnaswamy et al., 2017). Comparisons between surface EEG reconstructions and intracranial recordings have confirmed that source estimation based on high-density scalp EEG correctly localizes the current source of electrical activity in deep structures (Fahimi Hnazaee et al., 2020; Luo et al., 2007; Nahum et al., 2011), thus giving credence to our results. Nevertheless, the role of striatum and MTL for motor learning should additionally be investigated with other methods.

In conclusion, network interactions, specifically in the alpha and beta band, are associated with performance in a wide variety of functions in healthy humans as well as patients with brain lesions, including movements, language, spatial attention (Dubovik et al., 2012; Guggisberg et al., 2015; Rizk et al., 2013), memory (Dubovik et al., 2012), and vision (Allaman et al., 2021). The present findings show that network interactions are crucial for learning as well, suggesting new opportunities for enhancement of learning that are applicable not only to motor learning, but to many, if not all, domains of human behavior. Additionally, from a therapeutic perspective, the present study suggests that training techniques should attempt to facilitate network interactions rather than inducing local motor activations. For instance, neuromodulation techniques such as brain stimulation (Nicolo et al., 2018; Polanía et al., 2012; Schwab et al., 2019) and neurofeedback (Mottaz et al., 2018) can enhance network interactions, and this can lead to improved performance.

## AUTHOR CONTRIBUTIONS

Branislav Savic: Supervision; Writing – original draft; Writing – review & editing. Anaïs Mottaz: Conceptualization; Data curation; Formal analysis; Investigation; Visualization. Leslie Allaman: Validation; Writing – review & editing. Adrian G. Guggisberg: Conceptualization; Funding acquisition; Investigation; Project administration; Supervision; Writing – original draft; Writing – review & editing.

## FUNDING INFORMATION

Adrian G. Guggisberg, Swiss National Science Foundation, Award ID: 320030_169275 and CRSII5-170985.

## TECHNICAL TERMS

Motor learning: The process through which individuals acquire and improve the execution of physical movements.
Event-related power changes: Changes in the EEG signal amplitude in response to specific stimuli or events (e.g., hand movement).
Functional connectivity: Statistical relationship between physiological signals in time used as index of neural interaction or communication between spatially separate brain regions.
Resting-state: State in which an individual is not explicitly engaged in a task.
Absolute imaginary component of coherency: Measure of functional connectivity quantifying the time-lagged synchronization between signals, thus avoiding spurious interactions due to volume conduction.
Weighted node degree: Graph theoretical measure indicating the strength of the connections between a node and all other nodes.
Memory consolidation: Set of processes stabilizing memory traces after the initial acquisition.
